# Supplementary material for: Failure mechanism of soft– hard- interbedded rock slopes in cold regions: Numerical simulation and theoretical analysis
Source: PLoS One. 2025 Mar 11;20(3):e0317299. doi: 10.1371/journal.pone.0317299 (PMC11896076; doi:10.1371/journal.pone.0317299)
Supplement: Attachment — Submitted filename: Response to Reviewers.docx [file pone.0317299.s002.docx]

Response to Reviewers

Reviewer#1
Comment 1: Purpose of this study is not given clearly (The authors have to indicate how to use results of this study in engineering practice). The authors should refer to novelty of the paper in the introduction section.
Revision explanation：Added description of how this is practised in real engineering situations,The specific modifications are:In the context of natural disasters endangering people's lives and property, the construction of prevention and control works can not be delayed, the use of UDEC software for numerical simulation of hazardous slopes can be obtained from specific hazardous areas, can be targeted prevention and control, prevention and control is more accurate.It also adds the novelty of the paper, specifically:The use of strength discounting for the simulation of different working conditions makes the numerical simulation easier, and it is only necessary to obtain the rock discount rate for the area.

Comment 2: The affiliation should include city, state/province (if applicable).
Revision explanation：The unit contains the city, e.g. Urumqi, China.

Comment 3: Manuscript text should be double-spaced.
Revision explanation：The line spacing of the article is in the format specified by the journal used.

Comment 4: The text contains quite syntax and grammatical errors. As a reviewer I cannot correct all these errors. The manuscript should be carefully edited by a native English Speaker and some parts of text should be written again. I have highlighted some errors.
For example, in Line 13 you should delete “A”. Furthermore, you should use the word “interbedded” instead of “Interceded”.
In Line 18, you should delete “-'” after natural and the sentence will become “freeze-thaw cycles, natural, and anchored surroundings”
In Line 23 without “the”.
In Line 25 do not repeat the word “research”.
In Line 28: what means “UDEC” ? You should explain it.
In Line 36: “the” with capital the first letter.
In Line 38: “Promotes” instead of “promote”
Lines 45 – 46 are confused. Write them with another way.
Line 53 “are” instead of “is”.
Line 61 “make” instead of “makes”
Revision explanation：

In line 13, ‘A’ has been deleted and ‘Interceded’ has been amended throughout the text and replaced with ‘interbedded’.

The ‘-’ in line 18 has been deleted;

The word ‘the’ in line 23 has been deleted；

In Line 25： ‘research’ has been deleted;

In Line 28: UDEC is a numerical modelling software, modified to read ‘UDEC software’；

In Line 36:It has been modified;

In Line 38:It has been modified;

Lines 45 – 46：The sentence has been reworded;

In Line 53:It has been modified;

In Line 61:It has been modified;

Comment 5: In Line 114: In Table 2 uniaxial compressive strength is not shown.
Revision explanation：Data on uniaxial compressive strength were added to Tables 1 and 2.

Comment 6: Line 158: “monitoring point 3” instead of “monitoring point 2”
Revision explanation：The data from the monitoring sites were proofread and the sentence in line 158 was reworded to read:The X-direction displacement remains relatively small, with a maximum of only 0.82 mm. Among them, monitoring point 2 has the smallest degree of change in x-direction displacement during the simulation of the geotechnical body.

Comment 7: Line 160: “monitoring points 2 and 1” instead of “monitoring points 2 and 3”
Revision explanation：In Line 160:It has been modified;

Comment 8: Line 186: “ the forces at each monitoring point in Figure 12b increase before gradually stabilizing”. Are you sure that it increases?
Revision explanation：The statements in Line 186 have been proofread and rewritten as follows:The forces at each of the monitoring points in Figure 12b show an overall increasing trend of.

Comment 9: In Lines 194-197, the sentence should be written better.
Revision explanation：The passage has been rewritten to be more descriptive.The specific modifications are:As shown in Figure 13b, the stress process of monitoring point 1 can be divided into three stages, the first stage is the initial stage, where the stress curve gradually increases. the second stage is the stability stage, where the stress curve gradually tends to equilibrium. the third stage occurs when the rock body occurs damage, the stress enters into the destructive stage, the stress curve appears to be steeply inclined decline, and the continuous decline until the slope is stabilized.

Comment 10: The references are not in the right format.
Revision explanation：Changes have been made in accordance with the requirements of the journal.

Comment 11: The figures are not in the right format.
Revision explanation：Changes have been made in accordance with the requirements of the journal.

Comment 12: The conclusion is brief in length. Write more sentences and more analytically. Also the conclusions should try to mention some form of practical application of the results. Or further research that can lead to a practical use.
Revision explanation：The conclusion was rewritten and more content was added for description.Specifically modified to:

(1)Through discrete element UDEC software simulation, it has been determined that freeze-thaw conditions significantly impact the stability of soft– hard- interbedded rock slopes. Under natural conditions, the soft– hard- interbedded rock slope remains relatively stable.

(2)The failure mechanism of the slope can be summarized as follows: Long-term weathering, freeze-thaw cycles, and other factors lead to differential weathering in the soft– hard- interbedded rock slope with significant height differences due to geological structure. This results in irregular unstable structural planes, leading to slope instability and failure. The instability mode of the slope is characterized by unloading, stretching, and sliding.

(3)To design a prevention and control project for this area, a model under freeze-thaw conditions is incorporated into the command flow of anchor rod simulations representing working conditions with anchor rods. Under these working conditions, the overall stability of the slope is maintained; measures involving anchor rods can be implemented to prevent and control collapsed slopes effectively forming a collapse-anchor structural system.

Reviewer#2
• The introduction should be strengthened with more recent references to support the research gap and the significance of the study. The number of references is inadequate.
Revision explanation：References have been added for the last 3 years.

• Explain more about how to measure strength values and the standard methods used for values in Tables 1 and 2.
Revision explanation：The test work in the table has been completed in the early stage.The origins of the test data in Table 1 and Table 2 are elucidated.The amendments are as follows:( The work of this part has been completed in the early stage, which can be seen in Reference [23]).

• Has the role of ice wedge pressure in rock joints been seen for modeling?
Revision explanation：In this simulation, the main consideration is to use the strength discount method, the rock strength after 30 times of freezing and thawing as the effect of ice wedge pressure on the rock slope, so that the numerical simulation of the project is simpler and more convenient to operate.

• The density of rock materials in natural state and freezing – thawing are considered the same in modeling. This is despite the fact that the rock must face a decrease in density after enduring freezing.
Revision explanation：After the preliminary experimental work, it was learnt that since the simulated rock side slopes are dense rocks like tuff, the mass and volume changes of this rock after freeze-thaw cycles are small, so the factor of rock density was not considered in this simulation.

• Provide more details about the numerical simulation methods.
Revision explanation：Added about the numerical simulation methodology, briefly depicting the command flow used for numerical simulation and the process carried out.The amendments are as follows:The simulation mainly uses the FISH language in the UDEC software to import the boundary of the slope calculation model, and then through the ‘block create’, ‘block group’, and other commands to calculate the established model. After that, we can use ‘block create’, ‘block group’, and other commands to fill the blocks, which are mainly filled or grouped by Tyson polygon mesh; and then we can use ‘block prop mat’, ‘prop mat’, ‘block contact’ and other commands to fill the blocks. The blocks are assigned values by ‘block prop mat’, ‘prop mat’, ‘block contact’, and other commands, and finally, the stability of slopes under different working conditions is calculated.

• Discuss the implications of the findings for slope stability and risk assessment.
Revision explanation：The influence of research results on slope stability and risk assessment is included in the discussion.The amendments are as follows:By simulating natural freeze-thaw and bolt conditions using UDEC software, the calculation model can be monitored to assess slope stability based on observed displacement and cracks. UDEC, a discrete element simulation software, enables the simulation of rock mass falling. In practical engineering applications, this allows for quicker assessment of dangerous rock mass stability in a specific area, facilitates risk evaluation, and enables timely prevention and control of unstable hazardous rock masses.

• Conduct a sensitivity analysis to assess the impact of key model parameters.
Revision explanation：This numerical simulation is mainly for the natural working conditions, freeze-thaw conditions and working conditions with anchors, this paper prefers the simulation of the working conditions and does not consider the influence of many factors, so the sensitivity analysis is not used in this paper.

• Clearly articulate the practical implications of the findings.
Revision explanation：Added description of how this is practised in real engineering situations,The specific modifications are:In the context of natural disasters endangering people's lives and property, the construction of prevention and control works can not be delayed, the use of UDEC software for numerical simulation of hazardous slopes can be obtained from specific hazardous areas, can be targeted prevention and control, prevention and control is more accurate.It also adds the novelty of the paper, specifically:The use of strength discounting for the simulation of different working conditions makes the numerical simulation easier, and it is only necessary to obtain the rock discount rate for the area.
