# Supplementary material for: Failure mechanism of soft– hard- interbedded rock slopes in cold regions: Numerical simulation and theoretical analysis
Source: PLoS One. 2025 Mar 11;20(3):e0317299. doi: 10.1371/journal.pone.0317299 (PMC11896076; doi:10.1371/journal.pone.0317299)
Supplement: Attachment — Submitted filename: Response_to_Reviewer#1_auresp_3.docx [file pone.0317299.s004.docx]

Reviewer#1
Comment 1: Despite the fact that you corrected a lot of syntax and grammar errors, there are some ones to correct.

The manuscript should be carefully edited by a native English Speaker and some parts of text should be written again.
Revision explanation：The statement of the article has been verified and modified.

Comment 2:In table 1 and 2, you write "pniaxiai" instead of "uniaxial"
Revision explanation:Replaced "pniaxiai" with "uniaxial".

Comment 3: In table 12, there is decrease of forces. Not increase. Otherwise, you should refer that the tensile stress increases. you should explain it better.
Revision explanation：Revise the original statement “Monitoring point 1 decreases and then increases” to read “The increase of monitoring point 1 is larger”.

Comment 4: The conclusions are brief in length. Write more sentences and more analytically. Also, the conclusions should try to mention some form of practical application of the results. Or further research that can lead to a practical use.

Revision explanation：Additional clarification of conclusion 1.New conclusion 4 added.
